# Investigation of the effects of phytogenic dietary additives on growth performance, nutrient utilization, economic efficiency and health of *Pangasius hypophthalmus* : implications for sustainable aquaculture development

**DOI:** 10.1038/s41598-025-03658-5

**Published:** 2025-07-02

**Authors:** Habib Ul Hassan, Amjad Ali, Basim S. A. Al Sulivany, Muhammad Bilal, Roohi Kanwal, Muhammad Ahsan Raza, Abdul Arslan, Meer Zeeshan Ijaz, Muhammad Kabir, Mohammad Rizwan Khan, Nadiah Wan Rasdi, Takaomi Arai

**Affiliations:** 1 Department of Zoology (MRC & RC), University of Karachi, Karachi, 75270 Pakistan; 2 Center of Excellence in Marine Biology, University of Karachi, Karachi, 75270 Pakistan; 3 Department of Biology, College of Science, University of Zakho, Duhok, Zakho, Iraq; 4 School of Molecular and Life Science, Curtin University, Perth, 6845 Australia; 5 Department of Biology, Government Postgraduate College Satellite Town, Gujranwala, Pakistan; 6 Department of Zoology, University of Sialkot, Sialkot, Punjab Pakistan; 7 Department of Biological Sciences, Thal University Bhakkar (University of Sargodha, Ex-Sub-Campus Bhakkar), Bhakkar, 30000 Punjab Pakistan; 8 Department of Chemistry, College of Science, , King Saud University, Riyadh, Saudi Arabia; 9 Faculty of Fisheries and Food Science, Universiti Malaysia, 21030 Kuala Terengganu, Malaysia; 10 Environmental and Life Sciences Programme, Faculty of Science, University Brunei Darussalam, Gadong, BE 1410 Brunei

**Keywords:** *Pangasius hypophthalmus*, Nutritional strategies, Biological performance, Cost-benefit analysis, Ecology, Zoology

## Abstract

Medicinal plants exhibit promising potential for the current needs in aquaculture production which is expected to grow in coming decades to feed a growing population and lead the blue revolution. Medicinal plants are a substitute for phytotherapy in treating fish disease outbreaks and enhance biological and growth performance providing a sustainable alternative to antibiotics and chemicals. A seventy-day trial investigated the active components of herbal extracts in improving fish health, survival, and growth of Thai pangus. Five diets were designed: T1 (Turmeric, 5%), T2 (Garlic, 5%), T3 (aloe vera, 5%), T4 (Mixed, 5%), and a control diet (T5). The fish were fed 30% dietary protein to apparent satiation four times per day. Two thousand individuals were stocked in each tank, with two replicates for each treatment, and an average body weight of 0.2 ± 0.04 g. The physicochemical parameters of tank water remained within the optimum range. The highest final body weight (42.22 ± 0.56), weight gain (42.01 ± 0.82), specific growth rate (7.57 ± 0.02), survival rate (100%) and overall health was observed in T4 with (Mixed, 5%), extract, which was significantly different from the other treatments (*p* < 0.05), although treatments T1, T2, and T3 also showed improvements compared to the control group, which displayed significant different. The lowest FCR was recorded in T4 (0.8 ± 0.02) which was significantly different compared to other treatment groups (*p* < 0.05).The T4 enhanced the viscerosomatic index (4.86 ± 0.21) hepatosomatic index (2.16 ± 0.01) and condition factor (0.98 ± 0.05) (*p* < 0.05). The blood parameters of *Pangasius hypophthalmus* across all mixed medicinal plant concentration showed significant differences (*p* < 0.05). The study concludes that medicinal plants are effective nutritional supplements for improving the development and health of *P. hypophthalmus* under hatchery conditions. They represent a valuable alternative to antibiotics and providing effective, safe, and eco-friendly solutions for sustainable aquaculture.

## Introduction

The world population is rapidly increasing and is projected to reach 9.8 billion by 2050, leading to a doubling in the demand for food from aquatic sources also emerging need of a blue revolution and ensure long-term aquatic food production that meets societal challenges and viability^1,2^. A “Blue Revolution” focusing on sustainable aquatic food production and innovative technologies^1^. Food security and sustainable aquaculture stands as one of the extreme challenges of our time^1–3^. Aquaculture plays a crucial role in maintaining the food and nutritional stability of aquatic species while minimizing the overexploitation of wild stocks and preventing ecological degradation^2^. The sustainable aquatic food industry will lead in this endeavour once the existing aquaculture obstacles are overcome through innovation and improved regulations to increase the whole contribution to ecosystem protection^1,2^.

The *P. hypophthalmus*, commonly known as Pangasius is one of the most commonly farmed fish species for aquaculture that plays a pivotal role in providing food, nutrition, income, and livelihoods and its resilience ability to thrive in harsh ecological conditions^4^. This species has a wide range of physiological tolerances and can feed at the lowest trophic levels, making it particularly suitable for farming. It has high market demand, economically efficient, sustainability and low environmental impact^4,5^. It has a short production cycle, with fish reaching market size in 8–12 months due to its rapid growth rate reaching 1 to 1.5 kg. Pangasius is omnivorous and can thrive in high-density stocking conditions, as well as in environments with low dissolved oxygen and low salinity. Pangasius is a key fish species that provides economic opportunities for farmers and a dependable, nutritious product for consumers globally. Nutritional profiles for pangasius can vary across different sources. However, a 100 g serving of pangasius fillet typically contains approximately 17 g of protein, 5.6 g of fat .Additionally, this fish offers a high protein content, contains iron, has low fat, and has potential medicinal value^5–8^. *Pangasius hypophthalmus* is a good source of omega-3 fatty acids, which may contribute to cardiovascular health, making it a heart-healthy food choice. These fatty acids can help lower “bad” LDL cholesterol levels and reduce the risk of cardiovascular diseases when included in a balanced diet. Moreover, they can help decrease inflammation, lower blood pressure, and improve overall cholesterol levels.

This fish is also an excellent source of high-quality protein, essential for muscle repair, growth, and overall health. Furthermore*, P. hypophthalmus* is rich in collagen, which is often used in supplements aimed at enhancing skin elasticity, joint health, and wound healing^4–6^.

In terms of vitamins and minerals, this fish contains important nutrients like vitamin D and vitamin B12, as well as minerals such as phosphorus and selenium. These nutrients support immune function, bone health, and overall well-being^7^. Pangasius also contains small amounts of antioxidants like selenium, which help protect cells from oxidative damage. Advancements in aquaculture nutrition and feed research are necessary to secure healthy diets for both fish and humans^1,2^. Proper nutritional consumption is essential for maintaining health and preventing chronic disease^1,2^. There is a greater need than ever for high-quality protein, making it imperative to find items that may boost fish growth, survivability, and production efficiency. Sustainable aquaculture operations depend on managing fish nutrition for optimal development and health, which helps minimize expenses and maximize growth performance in an eco-friendly way^9^. Due to rising aquaculture costs and demand, fish meal production is currently in limited supply worldwide^10^. In Pangasius culture, feed costs constitute 70–90% of expenses, potentially leading to loss or narrow profit margins^11^. Improving feed nutrient use is one way to increase aquaculture productivity and sustainability^1^. Enhancing feed quality and designing feeding plans to distribute and utilize nutrients efficiently can boost growth, feed conversion, survival, and disease prevention^12,13^. Recent studies have shown that herbs, as safe and environmentally friendly food additives, enhance immune function, growth performance, and survival^14^. The supplementation of plant extracts for optimal growth performance in some fish species has gained significant attention in aquaculture^15,16^. Plant extracts benefit fish by enhancing growth, survival, and protein content, regulating blood parameters, improving biological performances, and providing aphrodisiac, anti-stress, anti-pathogen properties, and better feed consumption^17,18^. *Curcuma longa*, known as turmeric, is a member of the Zingiberaceae family of medicinal plants. Turmeric’s main ingredients are curcuminoids, which include bisdemethoxycurcumin, desmethoxycurcumin, and curcumin. Curcuminoids give turmeric anti-inflammatory, antibacterial, anti-platelet, anti-fungal, neuroprotective, hypoglycemic, antiviral, antioxidant, anticancer, and cholesterol-lowering properties^19^. Research has demonstrated the applicability of herbs like turmeric as feed supplements in aquaculture, enhancing growth, survival, and feed conversion in species like goldfish, which is an ornamental fish, guppy, climbing perch, and rohu^13,20–23^.

Garlic and aloe vera are medicinal plants^24^ with immunostimulant effects^25^ promoting growth^26^ antioxidants, and antimicrobial benefits in fish^27^. Garlic has been shown to improve fish development, survival, feed utilization, and body composition^28^. *Aloe vera* has antiviral and antibacterial properties, and combining it with other herbal extracts could enhance benefits for fish. For example, thyme can improve the development^8^ health^12^ and resistance of *Sparidentex hasta* fry^8,29–31^.

Black seed (*Nigella sativa*) is a medicinal plant belonging to the Ranunculaceae family and has been recognized for its benefits for over 1400 years. Due to its numerous medicinal properties, black seed is widely cultivated and used across the globe. The pharmacological effects of this plant primarily stem from its seeds, which contain various bioactive compounds, including thymoquinone, thymol, nigellicine, and nigellidine^32^.

The rapid growth of fish aquaculture has led to numerous diseases, causing economic losses. One of the most common diseases identified in cultured organisms is bacterial disease produced by the pathogenic bacteria *Aeromonas hydrophilla*, which causes "Motile Aeromonas Septicemia" (MAS), particularly in freshwater fish species in tropical waters. The bacteria affect *P. hypophthalmus,* one of the most essential freshwater commodities, and can infect fish of all sizes^17,23^. Traditional antibiotics and chemicals are now banned due to drug residues, pathogen resistance, and environmental pollution^33^. Herb extracts have become promising supplements due to their effectiveness, safety, and environmental friendliness. The effectiveness of their constituents, polyphenols, polysaccharides, saponins, flavonoids, alkaloids, and essential oils, in treating bacterial, fungal, parasitic, and viral illnesses in fish is well documented. For example, black cumin oil and powder have antibacterial qualities against *A. hydrophila*, making them a promising herbal remedy for conditions like irritation, diabetes, hypertension, and asthma while improving fish species’ blood hemoglobin, hematocrit, and globulin levels^34,35^. Phytotherapy is now recognized as a successful adjunctive or alternative therapy^36^. Herbal medicines, known for their excellent therapeutic effectiveness, low toxicity, minimal side effects, variety of drug targets, and reduced likelihood of developing drug resistance, have been extensively used in fish aquaculture. They have emerged as a preferred therapy to replace antibiotics and chemicals^33^. Despite the potential benefits, no experiments have been conducted to investigate the effects of specific extract mixes in aquaculture. The primary goal of this trial was to examine how medicinal plant extracts affected growth performance, feed utilization and economic efficiency and disease resistance of *P. hypophthalmus.* However, studies suggest that combining herbal extracts with other natural products can amplify benefits. Therefore, the current study aims to analyze the impact of dietary inclusion of medicinal plant extracts, both single and mixed doses, on enhancing the biological performance, growth, biochemical composition, feed utilization, survival, and disease prevention in *P. hypophthalmus* for sustainable aquaculture.

## Materials and methods

### Ethical approval

The Aquatic Life Ethics Committee of the Sindh Fisheries Department and Fisheries Development Board authorized this research and methods were performed in accordance with the relevant guidelines and regulations. This study was conducted in accordance with the ARRIVE guidelines.

### Experimental design and rearing system

The study was conducted in the Sindh Thatta Keenjhar Backyard Hatchery (N: 24.895450, E: 68.054164) from 2022 to 2023. Healthy *Pangasius hypophthalmus* seed were procured from a Tawakkal Fish Hatchery in Punjab Pakistan and acclimatized one week before the study. Two thousand individuals were stocked in each tank, with two replicates for each treatment, and an average body weight of 0.2 ± 0.04 g. The dimensions of each tank were (length 548 cm × width 213 cm × depth 100 cm) and volume (11,672.4 L) each and provided with a steady supply of water and continuous aeration. The 5-dosage prepared for the trials T1 (Turmeric, 5%), T2 (Garlic, 5%), T3 (Aloe vera, 5%), T4 (Mixed 5%), and Control (T5). The mixed dose included all three medicinal plants: 5% turmeric, garlic, aloe vera, and 2% black seeds which were added in T1, T2, T3, and T4. Black seed (*Nigella sativa)* was purchased from a local shop in Karachi and grinded into powder form. The black seed powder into the basal diet. The black seed improves health and controls bacterial infection. The 30% protein diets were given until they were visibly satisfied. The fish were fed at four daily meal frequencies, and their body weight determined the rate for seventy days. The study consisted of a 70-day main experimental phase to assess growth and biological performance, followed by a 10-day post-experimental phytotherapy phase to evaluate the potential therapeutic effects of plant-based treatment. The growth monitoring was done on a weekly basis by randomly collecting seeds for each treatment and observing all growth, survival, biological, and feed consumption parameters. The unused feed and other debris were drained out of the tank following each feeding. The process of collecting fresh excrement involved siphoning it into a circular basin. Every two days, 25% of the water volume was replaced to provide the highest possible level of water quality.

### Feed and herbs preparation, formulation, and experimental diets

Easily accessible components such as bone meal, dried fish meal, soybean oil, rice bran, wheat bran, wheat flour, turmeric, garlic, aloe vera, and black cumin, as well as vitamins and minerals, were sourced from the local market. Four varieties of medicinal plant extracts were used for the experimental work, including turmeric, garlic, aloe vera and mixed dose. To prepare the herbs for each treatment, the feed and herb ingredients were milled into a powder, weighed, and thoroughly mixed. A mixed dough, including water and oil, was added to create small-sized powder and pellets. As for the feed pellets, drying should be done before packaging them in plastic bags and not vice versa. They should be stored at − 15 °C until needed^37^. The prepared meals were tested for dry matter, crude fat, crude protein, moisture, and crude oil content. Table 1 outlines the preparation of the five experimental doses: T1 (turmeric, 5%), T2 (garlic, 5%), T3 (aloe vera, 5%), T4 (mixed, 5%), and the Control (T5).

**Table 1:** Constituents of the trial diets on a dry weight basis Kg^1^ and the proximate (%) composition of the research diets.

Ingredients (%)	Investigational diets				
	(T1)	(T2)	(T3)	Mixed (T4)	(T5) ©
Fish bone meal	12	12	12	12	12
Fish meals	30	30	30	30	30
Soybean meals	15	15	15	15	20
Rice bran	10	10	10	10	10
Black seed	2	2	2	2	–
Bread flour	12	12	12	12	12
Corn gluten	10	10	10	10	10
Medicinal plants extracts	5 (Turmeric)	5 (Garlic)	5 (Aloe vera)	5 (T + G + A)	–
Vitamin and minerals	2.5	2.5	2.5	2.5	2.5
Fish protein hydrolysate	1.5	1.5	1.5	1.5	1.5
Total (%)	100	100	100	100	100
Chemical analysis					
Crude lipids	8.6 ± 0.2	8.6 ± 0.2	8.4 ± 0.2	8.6 ± 0.5	8.2 ± 0.1
Crude proteins	30.61 ± 0.2	30.62 ± 0.2	30.71 ± 0.2	30.81 ± 0.4	30.22 ± 0.3
Crude fibers	8.1 ± 0.2	8.2 ± 0.8	8.8 ± 0.2	8.8 ± 0.3	8.0 ± 0.2
Moisture	10.1 ± 0.2	10.1 ± 0.1	10.4 ± 0.2	10.4 ± 0.1	9.5 ± 0.4
Ash	9.5 ± 0.2	9.5 ± 0.4	9.4 ± 0.4	9.6 ± 0.5	9.2 ± 0.2

### Growth survival and feed conversion parameter

The biotechnical parameters were calculated according to Hassan et al.^37^.

Average daily weight gain (ADWG, g) = (Final body weight–Initial body weight)/days.

Weight gain (WG, g) = Final body weight (g)—Initial body weight (g).

Feed conversion ratio (FCR) = Feed intake (g)/Weight gain (g).

Protein efficiency ratio (PER): Net weight gain /Protein in feed.

Survival (%) = (No of fish survival/No. of fish stocked) × 100.

Specific growth rate (SGR, %/day)) = LnW_2_ − Ln W_1_/T_2_ − T_1_ × 100.

Where W_2_ = Final body weight (g) at time T_2_ and W_1_ = Initial body weight (g) at time (days) T_1_.

### Water quality monitoring

The physicochemical parameters, including temperature, dissolved oxygen (DO), and pH, were recorded daily using a multi-parameter device (HI-98194). According to Hassan et al.^38^, ammonia, nitrate, and nitrite levels were measured weekly using chemical methods.

### Biochemical analysis and organsomatic indices

At the trails, 10 fish were collected from each tank, and carcass samples were collected, according to Hassan et al.^38^. We collected five fish from each treatment from each treatment to determine the organ somatic parameters like HSI, VSI, and CF^39^. The gross energy of the diet was calculated according to the methods of NRC^40^, the methods used for considering note of the ash, moisture, crude protein, and crude fat contents.

Hepatosomatic index (HSI, %): [Liver weight (g) /Body weight (g)] × 100.

Viscerosomatic index (VSI, %): [Visceral weight (g)/ Body weight (g)] × 100.

Fulton’s condition factor (CF): [FBW (g)/ total length cm^3^] × 100.

### Diseases diagnosis and management

After completing the trials, 210 live infected fish were collected from each treatment group. According to the methods of Noga^41^, the ten fish collected were dissected to obtain blood samples. The blood analyses were conducted according to the protocols established by Noga and Wang et al.^41,42^. Under sterile conditions, bacterial swabs were taken aseptically from the kidneys, liver, spleen, skin, and any eye lesions using a sterile loop. According to Wang et al.^42^, isolation of bacteria, Trypticase Soy Agar (TSA), and Saboraud Dextrose Agar, respectively. The diluted sample was plated over a sterilized nutrient agar medium and incubated at 37 °C for 24 h. The *P. hypophthalmus* with petechial hemorrhages were obtained for bacteriological analysis. Bacterial identification was achieved through macroscopic tests, Gram staining, and biochemical tests. *Pangasius hypophthalmus* samples were analyzed for size, shape, and pigmentation. All the impacted fish were collected from the each treatments and stocked separate tank and applied the mixed feeds dose (5% Turmeric + Garlic + Aloe vera) for the treatment of bacterial infection and hemorrhaged fish. After the completetion of ten days collected 10 fish from the treated fish for haematological assessment^36^.

### Statistical analysis

The normality test revealed that the data were normally distributed, and analysis of variance (ANOVA) was utilized to evaluate the data (SPSS, 2007). Using the Duncan-Waller approach, the differences between individual means were assessed at a significant level (*p* < 0.05).

## Results

### Water quality parameters

The findings of water quality parameters are provided in Table 2. The means (± standard deviation) of all parameters did not differ significantly between treatments. The temperature, DO, pH, ammonia, alkalinity, and nitrate were optimum for fish rearing.

**Table 2:** Water physicochemical parameters (Mean ± SD) of each treatment during the experimental period.

Physicochemical parameters	Treatments				
	(T1)	(T2)	(T3)	(T4)	(T5)
DO (mg/l)	6.28 ± 1.85	6.26 ± 1.74	6.24 ± 1.82	6.21 ± 1.71	6.21 ± 1.66
Temperature (°C)	29.02 ± 1.14	29.01 ± 1.82	29.04 ± 1.66	29.02 ± 1.71	29.04 ± 1.72
Salinity (ppt)	0.2 ± 0.06	0.2 ± 0.08	0.2 ± 0.04	0.2 ± 0.05	0.2 ± 0.07
Nitrite (mg/l)	0.012 ± 0.005	0.012 ± 0.004	0.012 ± 0.006	0.012 ± 0.001	0.013 ± 0.002
pH	7.20 ± 0.84	7.14 ± 1.01	7.11 ± 7.11	7.10 ± 1.22	7.12 ± 1.02
Nitrate (mg/l)	1.70 ± 0.24	1.81 ± 0.40	1.82 ± 0.45	1.82 ± 0.56	1.83 ± 0.67
Alkalinity (mg/l)	92.6 ± 27.54	91.6 ± 30.08	91.4 ± 33.02	91.6 ± 32.02	91.8 ± 30.41
Turbidity (NTU)	1.64 ± 0.15	1.64 ± 0.24	1.63 ± 0.44	1.68 ± 0.12	1.60 ± 0.18
Ammonia (mg/l)	0.011 ± 0.001	0.011 ± 0.002	0.041 ± 0.003	0.012 ± 0.004	0.012 ± 0.004

All monitored water quality parameters remained within the optimal range for the cultivation of *Pangasius hypophthalmus* throughout the experimental period.

### Growth, feed conversion, survival and Organsomatic indices

**Fig. 1:**
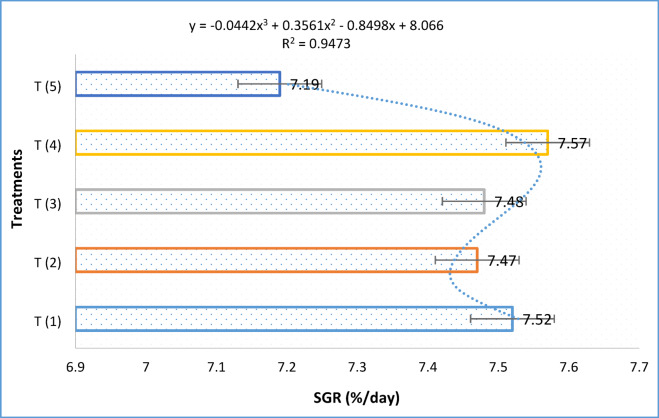
The polynomial regression analysis of specific growth rate (%/day) relative to dietary additive dosage showed a significant relationship, indicating the optimal inclusion level for maximizing growth performance in *Pangasius hypophthalmus*.

Table 3 shows the results of the extract herbs, which showed that the growth performance of Thai pangus changed greatly depending on the dietary inclusion. The herbal supplementation of mixed 5% (T4) had the greatest FBW, FBL, LG, WG, SGR, and ADWG, followed by feeding of T1, T2, and T3, while the control group without herbs extract had the lowest FBW, FBL, LG, WG, SGR, and ADWG. Table 3 indicated significant variations in survival rate in the treatments; T1, T2, T3, and T4 had the highest survival rate, followed by T5, which is recorded, in Table 3. The SGR carving reached the maximum point at T (4) and showed that determination coefficient (r^2^) = 0.95. The polynomial regression indicated that the (T4) is a sustainable dose for the Thai pangus growth and biological performance as shown in Fig. 1.

**Table 3:** Morphological and biological performance and survivability of *Pangasius hypophthalmus* under the different medicinal plant extract.

Biotechnical parameters	Treatments				
	Turmeric (T1)	Garlic (T2)	Aloe vera (T3)	Mixed (T4)	Control (T5)
IBW (g)	0.21 ± 0.04^a^	0.21 ± 0.04^a^	0.21 ± 0.04^a^	0.21 ± 0.04^a^	0.21 ± 0.04^a^
FBW (g)	40.81 ± 0.24^a^	39.22 ± 0.53^b^	39.61 ± 0.44^b^	42.22 ± 0.56^a^	32.24 ± 0.31^c^
FBL (Lf,cm)	15.16 ± 0.11	14.04 ± 0.21	14.02 ± 0.44	16.24 ± 0.23	13.62 ± 0.42
ADWG (g)	0.57 ± 0.01^a^	0.56 ± 0.01^b^	0.56 ± 0.02^b^	0.60 ± 0.04^a^	0.46 ± 0.01^c^
WG (g)	40.59 ± 0.23^a^	39.01 ± 0.53^b^	39.41 ± 0.11^b^	42.01 ± 0.82^a^	32.04 ± 0.24^c^
SGR (%/day)	7.52 ± 0.01^a^	7.47 ± 0.04^b^	7.48 ± 0.01^a^	7.57 ± 0.02^a^	7.19 ± 0.01^c^
FCR (g)	0.8 ± 0.01^a^	0.9 ± 0.03^b^	0.9 ± 0.01^b^	0.8 ± 0.02^a^	0.94 ± 0.02^b^
PER	1.35 ± 0.01^a^	1.30 ± 0.01^b^	1.31 ± 0.02^b^	1.40 ± 0.01^a^	1.06 ± 0.03^c^
CF (%)	1.17 ± 0.02^a^	1.41 ± 0.01^a^	1.43 ± 0.04^a^	0.98 ± 0.05^b^	1.27 ± 0.04^a^
HSI (%)	2.14 ± 0.14^a^	2.25 ± 0.34^b^	2.44 ± 0.06^b^	2.16 ± 0.01^a^	2.10 ± 0.01^a^
VSI (%)	5.81 ± 0.21^a^	5.82 ± 0.34^a^	5.66 ± 0.06^b^	4.86 ± 0.21^a^	4.44 ± 0.41^b^
SR (%)	99.0 ± 0.06^b^	98.2 ± 0.08^b^	98. ± 0.04^b^	100 ± 0.00^a^	90 ± 1.21^c^

The data indicates the mean ± SD of fish, with substantial variances in each row with different superscripts (*p* < 0.05).

**Table 4:** Final whole body proximate composition (% wet weight) of experimental *Pangasius hypophthalmus* at different medicinal plant extract for 70 day trial.

Parameters (%)	Treatments				
	(T1)	(T2)	(T3)	(T4)	(T5)
Crude protein	30.22 ± 0.41	30.12 ± 0.44	30.50 ± 0.12	30.72 ± 1.4	30.42 ± 1.4
Moisture	72.40 ± 0.04	72.20 ± 1.11	72.60.1 ± 0.31	72.52 ± 0.02	72.52 ± 0.02
Crude lipid	7 .20 ± 1.5	7.42 ± 1.60	7.68 ± 1.80	7.62 ± 1.44	7.44 ± 1.44
Ash	9.72 ± 1.5	9.81 ± 1.40	9.77 ± 1.60	9.66 ± 1.67	9.64 ± 1.66

The significant variations were observedin the Condition Factor (CF), Hepatosomatic Index (HSI), and Viscerosomatic Index (VSI). The CF values in the treatment groups did not show significant differences. In terms of HSI there were no significant differences among treatments T1, T2, and T3, which all had notably higher values compared to T4. A substantial positive correlation was observed between VSI values and herbal supplementation, with the highest VSI recorded in T1,T2, T3, while the lowest VSI values were seen in treatments T4 and T5.

### Carcass compositions

Table 4 shows the proximate composition of the carcass as measured by wet weight. Feeding treatments had no significant effect on the carcass’ moisture and protein content. The carcass’s lipid and ash content, on the other hand, varied dramatically across treatments. The carcasses showed a favorable correlation.

### Diagnosis of diseases and management

After the 70 days 10 live fish were randomly collected from each treatment. After the proper morphological study of collected fish from each treatment, the hematological and pathological investigation was done on the affected fish as shown in Table 5. The petechial hemorrhages of fish individuals were dissected, and samples of the organs were obtained for bacteriological analysis.

**Table 5:** The evaluation of blood parameters in infected fish before and after phytotherapy.The values are presented as mean ± SD. Bars without common superscripts differed significantly (p < 0.05).

Blood parameters	Treatments	
	1st phase, infected fish	2nd phase, after phytotherapy
Hemoglobin (g dL^−1^)	6.7 ± 0.07^b^	12.84 ± 0.06^b^
Neutrophils %	1.12 ± 0.98^b^	3.1 ± 0.55^b^
Lymphocytes %	88.12 ± 0.88^b^	89.8 ± 0.81^a^
Monocytes %	8.56 ± 0.64^a^	4.8 ± 0.88^b^
Eosinophil’s %	1.88 ± 0.60^a^	0.5 ± 0.88^b^
Platelets (cells/µL)	40,000.22 ± 2.30^a^	550,000.4 ± 1.043^a^
Total RBC (10^6^ µL^−1^)	1.22 ± 0.04^a^	2.22 ± 0.04^a^
HCT (%)	24.80 ± 0.30^a^	32.02 ± 0.20^a^
MCV (fL)	134.9 ± 0.54^a^	140.65 ± 0.66^b^
MCH (pg)	26.4 ± 0.66^a^	24.88 ± 0.56^a^
MCHC g/dL	24.21 ± 0.24^a^	34.3 ± 0.16^a^
WBC Cells/mm^3^ (µL)	130,000 ± 12.94^b^	80,000 ± 10.52^c^
Albumin(g/L)	1.15 ± 0.24^a^	2.1 ± 0.23^a^
Globulin(g/L)	4.52 ± 0.99^a^	2.8 ± 0.99^a^
Total protein(g/L)	5.84 ± 0.88^a^	4.6 ± 0.96^b^

The fish with bacterial septicemia moved slowly, had low vitality, and consumed less food, and their body surface was congested to varied degrees. The affected were dissected, and watery blood was found in the belly cavity. In addition, their gills were swollen and pale yellow. The bowel contained a tiny amount of food and had minimal congestion, while the kidney was substantially congested. *Aeromonas hydrophilla* bacteria grow on spread and streaked AIM plates, indicating that they were largely connected with ulcerative lesions on *P. hypophthalmus*. The isolates produced yellowish, opaque colonies on TSA agar. Bacteria were recovered from the fish organs. After 48 h of growth, the dominant bacteria were identified as colonies of the same color and form that occupied the greatest proportion of the total area of the culture media. The colonies were flat, elevated, and circular. Microscopic testing was performed by staining Gram, analysing the color and shape of bacteria, and doing biochemical assays. The outcome of their comparative morphological, biochemical, and physiological testing.

### Haematological assessment

The results of the hematological parameters for *P. hypophthalmus* are presented in Table 5. The infected fish reduced the hemoglobin, neutrophils, RBC, HCT, MCV, MCHC, albumin but increase the Monocytes, Eosinophil’s, MCH, WBC and Globulin. The blood parameters of *P. hypophthalmus* across all mixed medicinal plant concentration showed significant differences (*P* > 0.05). *Pangasius hypophthalmus* that were fed a (5%) mixed diets containing of turmeric, garlic and aloe vera feed exhibited the significance effects on blood parameters (see Table 5).

### Bacterial infection (red spot and dropsy diseases)

The results indicated the bacterial infection *Aeromonas hydrophilla*, which causes "Motile Aeromonas Septicemia" (MAS) disease. It is a septicaemic condition known as Motile Aeromonas Septicemia (MAS). The infection occurs in fish and during the grow-out phase.

#### Aetiology (Causative agent)

Motile aeromonas, especially *Aeromonas hydrophila*, cause the disease condition.

#### Risk factors

This infection occurs during changes in weather, from dry to the rainy season, especially if fish are stressed by handling and transportation. Overcast sky with intermittent rain causing a lack of sunlight and wide fluctuations in water temperature may predispose fish infection and disease outbreaks.

#### Clinical signs

After the proper investigation was done, we observed the symptoms and signs of the fish, including accumulation of red fluid in the body cavity and destruction of liver cells, green or yellow coloration of the liver and slow swimming, anorexia, petechial hemorrhages on the body, head, mouth, and base of the fins. The behavioral differences, as well as swimming, were observed. Abnormal position, cease feeding, or refuse food intake. Changes in body physique, shape, and color, such as discoloration of the body. Fin edges become pale or reddish, and scales are removed. Water or reddish fluid accumulates in the body and fin roots. Sudden movement and jumping from the water, rubbing the body against rough. Movement along one’s axis, backward or forward, tail down or head down, oblong, vertical or horizontal, with an imbalanced body. Exophthalmos or enophthalmos and impaired eyesight.

Five individuals were investigated for fluid buildup or renal failure. The accumulation of bodily fluids leads to fish bloating, protruding skin, swollen bellies, and haemorrhagic ulcers that occur on the skin and fins.

#### Phytotherapy treatments

Fish aquaculture suffers substantial financial losses and a higher death rate due to bacterial infections, which the majority of farmed fish suffer from. Antibiotics are commonly used together to treat fish bacteriosis. Nevertheless, significant issues like drug residues, drug-resistant strains, and environmental contamination have been brought on by the misuse of antibiotics. Thus, the secure and efficient means of treating bacterial infections in fish. Our study indicated that the dietary additive groups like T1, T2, T3, and T4 significantly affect fish health and survival. The disease outbreak was observed in the control group. The fish outbreak bacterial infection was observed in control groups. Then, all 200 infected fish of the allgroups were transferred to the separate tank and applied mixed feeds dose (5% T + G + A) for a further 10 days (post-experimental phytotherapy phase). The fish recovered from the infection using the medicinal plant are shown in Table 5.

#### Prevention

The rapid growth of fish aquaculture has been driven by increasing demand. Still, it has also brought challenges, such as the emergence of various diseases and environmental degradation, leading to significant financial losses in the industry. While pesticides and antibiotics are commonly used to treat and prevent fish diseases, their use is increasingly restricted or banned due to concerns over medication residues, disease resistance, and environmental pollution. Phytotherapy has emerged as a promising supplement and alternative, offering low drug resistance, safety, effectiveness, and environmental friendliness. Natural treatments in fish health management primarily work through potent immunostimulation, antioxidant properties, or direct anti-pathogenic effects, making them a cost-effective and sustainable approach to disease prevention and management in aquaculture.

## Discussions

The health of fish is significantly affected by physicochemical characteristics, which are essential for their well-being^37^. In closed aquaculture systems, overfeeding leads to increased waste, degrading water quality by producing ammonia and depleting oxygen^43^. Consequently, inadequate water quality conditions reduce feed intake, increase the feed conversion ratio (FCR), and lower survival rates^44,45^. Quality parameters such as temperature, salinity, dissolved oxygen (DO), pH, ammonia, alkalinity, and nitrate varied somewhat among treatments. Still, they remained within the suitable range for *P. hypophthalmus* cultivation, as shown in Table 2. Although *P. hypophthalmus* has a broad temperature tolerance range (20–38 °C), the species grows most optimally between 24 and 34 °C^46^. Additionally, *P. hypophthalmus* prefers an ammonia level between 0.002 and 0.06 mg/L. Fish mortality increases when ammonia levels rise from 0.24 to 0.50 mg/L^47^. According to Kucuk^3^, the water quality results in this investigation generally support the growth of *P. hypophthalmus*. The study revealed that *P. hypophthalmus* could grow and survive within these parameters^4^.

After a 70-day feeding study, *P. hypophthalmus* fed diets supplemented with turmeric, garlic, aloe vera, and combined supplementation showed increased growth in comparison to the control diet. Conversely, fish fed a diet devoid of supplements had much worse growth and survival rates. Turmeric has been demonstrated to have a positive effect on growth performance in *P. hypophthalmus*^6^, African catfish *(Clarias gariepinus)*^48^ common carp *(Cyprinus carpio)*^49^ and rohu (*Labeo rohita)*^23^ When compared to the control diet, *P. hypophthalmus* that received feed supplements including turmeric also displayed enhanced growth and feed functionality, FCR, and intake^50^.

To the best of our knowledge, this is the first study to discuss including medicinal herbs in a fish diet. According to the current investigation, blended supplements may be a valuable herb for fostering *P. hypophthalmus* development^51^. *Osphronemus goramy* body weight rose when T1 and Mixed (T4) supplements were added to their food^52^.

Garlic, a medicinal plant with immunostimulant effects, contains growth promoters, antioxidants, and antimicrobials^25^ and ensures improved feed digestibility^27^. Research has shown garlic’s impact on *P. hypophthalmus* growth, survival, feed use, and proximate composition^26^, with various species experiencing growth, survival, and biological performance improvements^53^.

Among the herbs examined, the one used in this study (T2) was the least effective in promoting growth. Numerous research has shown that adding herbs to the *P. hypophthalmus* diet can improve feed performance and development^54,55^. When ginger (*Zingiber officinale*) and nutritional onions (*Allium cepa*) were added to tiger grouper (*E. fuscoguttatus*) formula feed, the fish showed noticeably improved SGR, feed efficiency, and survival rates as compared to the control group^54^.

In a similar vein, feeding orange-spotted grouper (*E. coioides*) garlic (*Allium sativa*) significantly enhanced the fish’s feed efficiency, development rate, and resistance to infection by *Streptococcus iniae*^55^. In a related study, after being fed katuk (*Sauropus androgynus*), orange-spotted grouper displayed a significant increase in weight gain and SGR^56^. These results suggest that grouper diets supplemented with the right kind of herbs may benefit from enhanced fish development and performance.

All of the treatment groups in the current study were in favor of adding herbs to the prepared menus. T2 and T3 had feed conversions that were much greater than T4 and comparable to T5, respectively. Nonetheless, the fish’s development was unaffected by the lack of supplementation. According to Huq^4^, the feed efficiency of T1 and T5 showed a significant overall improvement in terms of growth performance, PER, and FCR.

The *P. hypophthalmus* that were fed T4 meals exhibited the highest GP and the lowest feed consumption of all the treatments. This indicates that *P. hypophthalmus* development was stimulated by dietary supplementation with (T1) and (T4), without increasing feed intake, by boosting the efficiency of protein retention.

Additionally, the body protein content of T4 was marginally greater than that of T5, which further corroborated the *P. hypophthalmus* strong protein retention and efficacy. Previous studies on Japanese flounder^57^ and *Pangasius hypophthalmus*^58^ demonstrated that increased protein and protein retention efficiencies promote fish development.

According to the previous studies^52,54,56^ effects of medicinal plant extracts can vary significantly among different fish species. Excessive or poorly balanced supplementation can lead to toxicity, reduced palatability, or stress, especially if bioactive compounds are not dosed correctly. Additionally, uneaten feed or fish waste containing plant residues may negatively impact water quality or disrupt the microbial balance in the system if not properly monitored these findings were consistent with the previous observations^56,57,59^.

The use of medicinal plant extracts presents a promising strategy for promoting sustainable aquaculture, but it is essential to consider species-specific responses and potential environmental interactions.

This research offers a more comprehensive evaluation by assessing growth performance, nutrient utilization, health status, and economic efficiency specifically in *Pangasius hypophthalmus*. These findings support the broader application of plant-based feed additives as effective and eco-friendly alternatives to synthetic compounds in aquaculture systems^2,3,19^.

Furthermore, higher consumption of protein, gain, and retention all markedly accelerated the development rate of Nile tilapia^59^. Also, among the therapies, (T4) likewise had the best survival rate. This suggests that T1 and T4 are prospective herbs to enhance *P. hypophthalmus* growth, survival, and feed efficiency^3^.

Also, T1 and T5 had considerably greater CP, fat, and dry matter than T5. This showed that while the feed efficiency of *P. hypophthalmus* given mixed (T4) and turmeric (T1) was comparable to that of the standard diet, the addition of these herbs improved the digestion and absorption of nutrients. This finding is in line with a report on *P. hypophthalmus* in which the supplement of turmeric to the diet upgraded and altered the gut flora, leading to better growth, increased microbial enzymatic activity, increased numbers of beneficial bacteria, and increased nutritional digestibility^50^. Turmeric has also been reported to be a helpful digestive stimulant that promotes the activity of enzymes like lipase, chymotrypsin, and amylase^60^. It has been noted that incorporating herbs into peppermint (*Mentha piperita*) meal may enhance feed utilization and growth rate for fish species such as Caspian white fish (*Rutilus frisii kutum*) and Asian sea bass (*Lates calcarifer*)^61^.

This study assessed the physical status of *P. hypophthalmus* using the CF, HSI, and VSI. The fish fed (T1) and T4 had the highest HSI, but overall, there was no appreciable variation in HSI between the groups and control diets. Between the three therapies, there were no appreciable differences in the VSI or CF. the results pertaining to HSI support those reported by^62^.

There were no appreciable variations in HSI values between the treatment and control groups as a result of using medicinal plants. Furthermore, the present study demonstrated no discernible variation in the whole-body proximate composition between the fish-fed turmeric (T1) and T4), T2, T2, and the control group.

According to Patel^6^, *P. hypophthalmus* administered turmeric (T1) and mixed (T4) may have high body protein and fat levels in their carcass composition due to good growth and feed performance, body indices, and CP. Turmeric, a medicinal plant, is used as a coloring agent and spice. Its main ingredients are curcuminoids^19^, which have anti-inflammatory, antibacterial, and neuroprotective properties^63^. Because it increases growth survivability and feed conversion in a variety of fish species, turmeric’s growth-promoting qualities make it suitable and fit for aquaculture^13,20^.

*Aloe vera*, a tropical plant, has pharmacological activities like antiviral, antibacterial, and wound-healing effects^29^. However, its immunological effects on warm-blooded animals and its anti-toxicity and immunogenicity are not well understood^30^. Studies on *Aloe vera* extracts in aquaculture suggest that their benefits may be amplified when combined with other herbs or natural products (For example, thyme has been shown to enhance fish health and resistance^8^.

The fast growth of fish aquaculture has led to numerous diseases, causing economic losses, and traditional antibiotics and chemicals are now banned due to drug residues, pathogen resistance, and environmental pollution, which have been investigated by Adelmann^33^. Medicinal plants have become promising supplements due to their effectiveness and safety for sustainable aquaculture. Herbal extracts have been extensively studied for their efficacy in fish bacterial, fungal, parasitic, and viral diseases^36^. According to Aly^35^, black cumin oil and powder have antibacterial qualities against *A. hydrophila*, making them a promising herbal remedy for a number of conditions, such as inflammation, diabetes, hypertension, and asthma. They also help fish species’ blood hemoglobin, hematocrit, and globulin levels^35^.

The inclusion of plant-based feed additives in fish diets significantly affects fish health, growth, and economic efficiency through several key mechanisms. Bioactive compounds found in these plants, such as flavonoids, alkaloids, and essential oils, stimulate digestive enzymes, leading to improved nutrient absorption and a better feed conversion ratio (FCR). This results in enhanced growth performance and more efficient feed utilization complement the findings^3,27,50^ the medicinal plant enhanced the blood parameters. Phytotherapy has emerged as a promising alternative to antibiotics for treating bacterial infections in *P. hypophthalmus*. It utilizes the antimicrobial, immunostimulatory, and anti-inflammatory properties of various medicinal plants. Compounds such as allicin found in garlic (*Allium sativum*), curcumin from turmeric (*Curcuma longa*), and aloe vera (*Aloe vera*) demonstrate strong antibacterial effects, particularly against pathogens like *A. hydrophila*, which is a common cause of infection in Pangasius .The hematological parameters in the current research demonstrated significant alternation between the fish in the control group and other groups, which seems to be consistent with the results of our study^6,12,30^.

Phytotherapy has gained recognition as an effective treatment method^36^. An experiment using extracts from aloe vera, garlic, and turmeric was conducted to treat diseased fish, resulting in the recovery from bacterial infection^64–66^. Medicinal plant are commonly employed to treat illnesses and prevent diseases in humans and animals^67,68^. Due to their excellent therapeutic efficacy, low toxicity, minimal side effects, diverse drug targets, and reduced likelihood of developing drug resistance, they have been extensively utilized in fish aquaculture in recent decades. They have emerged as a preferred therapy to replace antibiotics and chemicals^33,69^.

## Conclusion

The findings of this study hold promise for the development of artificial feed with adjustable compositions to enhance *P. hypophthalmus* production in Pakistan’s aquaculture sector in a sustainable manner. Over a 70 days trial the *P. hypophthalmus* was exposed to five distinct treatment groups revealing that a blend of medicinal plant extracts (T4) resulted in the highest fish growthperformance, better feed utilization, and overall enhanced health compared to the other groups and making it the most effective diet. Additionally, a 10-day post-experimental phytotherapy phase confirmed the therapeutic effectiveness of the extracts, as indicated by improved recovery and reduced bacterial load in infected fish. These findings emphasize the dual role of medicinal plants as both growth promoters and natural alternatives to antibiotics in aquaculture. Incorporating bioactive plant compounds into fish diets presents a sustainable and eco-friendly strategy for improving fish health, reducing disease outbreaks, and supporting antibiotic-free aquaculture practices.

However, as aquaculture operations expand and environmental conditions deteriorate, various diseases have emerged, leading to significant financial losses. Phytotherapy has emerged as a viable alternative and supplement, offering low drug resistance, safety, effectiveness, and environmental compatibility. Herbal treatments are valued for their immune-boosting, antioxidant, and direct anti-pathogenic properties, presenting a cost-effective alternative for managing fish health. Further research is needed to test the efficacy of different fish species and life stages to determine species-specific responses and general applicability in commercial aquaculture. Additionally, explore the synergistic effects of combining various medicinal plant extracts or integrating them with probiotics.

## Data Availability

The data presented in the study are available on request from the first and corresponding author. The data are not publicly available due to the thesis that is being prepared from these data.
